# Building diverse participatory research teams in falls prevention: a qualitative interview study

**DOI:** 10.1186/s12877-026-08686-y

**Published:** 2026-11-10

**Authors:** Elisa-Marie Speckmann, Laura Himmelmann, Tim Stuckenschneider, Tania Zieschang, Milena von Kutzleben, Kathrin Boerner

**Affiliations:** 1 Department for Health Services Research, Geriatric Medicine/ School VI- School of Medicine and Health Services, Carl von Ossietzky Universität Oldenburg, Oldenburg, Germany; 2 Department for Health Services Research, Prevention and Rehabilitation Research/ School VI- School of Medicine and Health Services, Carl von Ossietzky Universität Oldenburg, Oldenburg, Germany

**Keywords:** Falls, Participatory research, Older adults, Prevention

## Abstract

**Background:**

An important area of participatory research and the establishment of participatory research teams (PRT) is falls prevention, as falls pose significant risks not only to individuals but also to healthcare systems worldwide. However, little is known about how PRTs are conducted within falls prevention research. Therefore, this study aimed to identify key factors that facilitate participatory research in this context.

**Methods:**

The PRT was involved in two projects, focusing on falls prevention: iSeFallED and TRAIL. We conducted eleven semi-structured interviews with each member of the PRT (six patient representatives, five health service representatives). The interviews were transcribed and then analyzed using qualitative content analysis.

**Results:**

Heterogeneous methods, communication grounded in values such as mutual respect, opportunities for networking, knowledge exchange, and intrinsic motivation were identified as important facilitators.

**Conclusion:**

This study provides insights into key factors for implementing participatory research in falls prevention.

**Trial registration:**

TRAIL NCT06652828, https://clinicaltrials.gov/study/NCT06652828#more-information, registered on 21stOctober 2024, and iSeFallED DRKS00035322, https://drks.de/search/de/trial/DRKS00035322, Registered on 5th March 2025.

**Supplementary information:**

The online version contains supplementary material available at https://doi.org/10.1186/s12877-026-08686-y.

## Introduction

The involvement of stakeholders is a fundamental component in the development of complex interventions [1] and is gaining increasing consideration in funding frameworks and research strategies [2]. The involvement, often named as patient and public involvement (PPI), is commonly defined as “research that is done `with´ or `by´ the public, not only ´to´, ´for´ or ´about´ them” [3]. In healthcare, PPI is used in different contexts such as quality improvement and safety [4]. Patient involvement can range between advising, being an equal partner and being completely involved in decision making [5]. Within health services research, participatory approaches have become essential, as they facilitate the inclusion of diverse perspectives, enhance the relevance of research for both communities and researchers, and contribute to improved recruitment and the overall quality of research outputs [6]. Evidence from systematic and narrative reviews across different populations further supports the value of participatory approaches. For example, a systematic review on participatory methods in pediatric research highlights that involving children through age-appropriate participatory methods can strengthen partnerships between researchers and young participants and enhance the meaningful inclusion of their perspectives in research processes [7]. Similarly, research involving older adults demonstrates that participatory approaches can facilitate access to local knowledge and social networks and support the engagement of otherwise hard-to-reach groups, thereby enriching the knowledge generated in health research [8]. At the same time, the literature emphasizes that participatory research requires careful management of expectations, roles, and resources among all stakeholders in order to ensure meaningful participation and sustainable research processes.

In the context of falls prevention, the active engagement of individuals is particularly critical because of their lived experience [9]. Approximately 30% of older adults experience at least one fall each year [10]. Beyond physical injuries, such as hip or humerus fractures, falls often lead to concerns about falling, reduced physical activity, diminished capacity to perform daily activities, and, in many cases, social isolation [11]. These consequences extend beyond the individual, placing substantial burdens on caregivers and the healthcare system [12]. Given the projected demographic shift towards an aging population, both the incidence and the impact of falls are expected to rise, underscoring the urgent need for effective prevention strategies.

Although there is robust evidence supporting the effectiveness of strength and balance exercises for falls prevention, their translation into routine practice remains insufficiently defined. Population-based data indicate that many older adults do not regularly engage in activities that are known to reduce fall risk. For example, only around 8% of older adults in Australia report performing balance exercises at least twice per week [11], and approximately 25% of adults in Germany report participating in gymnastics or strength training within the preceding month [12]. While these figures refer to general physical activity rather than participation in specific falls prevention intervention, they illustrate the limited uptake of preventive exercise among older adults. This gap between evidence-based recommendations and actual behavior highlights the challenge of implementing effective falls prevention strategies in real-world settings. It raises the question of how such interventions can be designed and delivered in ways that better align with the preferences, daily routines, capabilities, and perceived barriers of older adults. Global guidelines on falls prevention emphasized the importance of adapting interventions to local contexts and available resources [13]. However, contextual adaptation should not be limited to structural or organizational factors; it should also include meaningful engagement with the target population. In this regard, patient and public involvement (PPI) offers a structured approach to integrating knowledge of older adults into the development, refinement, and evaluation of falls prevention strategies. By actively involving older adults with lived experience, interventions can be iteratively tailored to their priorities, motivations, and contextual constraints, while co-creation helps identify which components are feasible and acceptable in practice. Although substantial work has been undertaken in the broader field of user and patient involvement, its application within falls prevention research remains comparatively underexplored and insufficiently systematized. Strengthening the integration of PPI in this field may therefore contribute not only to more context-sensitive intervention design but also to improved uptake, adherence, and ultimately effectiveness of falls prevention strategies.

Stakeholders, including patients and health service representatives, can provide invaluable insights into the needs and priorities of the target population. Their involvement is particularly beneficial in ensuring that falls prevention measures are not only evidence-based but also aligned with the lived realities including the regional infrastructure, preferences, and functional capacities of older adults. Incorporating their perspectives early in the development process can lead to more appropriately designed and better-accepted interventions, ultimately enhancing effectiveness and quality of life.

One structured method of stakeholder involvement is the use of advisory committees, often referred to as participatory research teams (PRTs), which engage with researchers during regular meetings [14]. Factors contributing to the successful involvement of stakeholders include strong interpersonal relationships, in-depth communication [15], and consistent engagement through regular meetings [16]. However, limited knowledge exists regarding the implementation of PRTs in the specific context of falls prevention among older populations. In particular, the perspectives of diverse stakeholders – such as individuals with a history of falling and representatives from health and social care – on their involvement in participatory research processes remain underexplored. While factors such as effective communication, mutual trust, and structured formats are known to support stakeholder engagement, it remains unclear how these and other aspects are experienced by participants within the specific context of secondary falls prevention.

This qualitative interview study, therefore, aims to explore perceived barriers and facilitators to conducting participatory research in falls prevention. By examining the experiences, roles, and reflections of members of a PRT – including both individuals with lived experience and health service professionals – this study seeks to generate insights into how participatory methods can be effectively applied to co-develop relevant and acceptable interventions for older adults at risk of falling.

## Methods

### Design

We conducted eleven semi-structured telephone interviews with each member of a PRT. The PRT is involved in two ongoing research projects TRAIL (“Training at the Limit of Balance Control on a Perturbation Treadmill to Prevent Unrecovered Falls in Geriatric Patients With and Without Cognitive Impairment”, NCT06652828) and iSeFallED (“Tailored Interventions to Prevent Functional Decline After a Sentinel Fall Presenting to the Emergency Department – iSeFallED”, DRKS00035322). Detailed descriptions of the study designs have been published elsewhere [17, 18]. Both projects are conducted by researchers from the Division of Geriatric Medicine within the Department of Health Services Research at the University of Oldenburg and focus on falls prevention in older adults. The project iSeFallED aims to assess the feasibility, acceptability and efficacy of a secondary falls prevention program, which should then be implemented into clinical practice [18]. The aim of the TRAIL study is to investigate the effectiveness of a perturbation-based balance training in preventing unrecovered falls [17].

TRAIL (S-557/2024), iSeFallED (2024 − 192), and the PRT itself (2024 − 160) received ethical approval from the Medical Ethics Committee of the Carl von Ossietzky University of Oldenburg and adhere to the principles outlined in the Declaration of Helsinki. The PRT was established in October 2024. It builds upon a previous PRT, conducted as part of the SeFallED study [19], with four of its former members participating in both the past and current PRT.

The PRT mets with researchers of both projects approximately every six weeks to discuss topics and provide feedback. To simplify, we refer to the researchers employed in the TRAIL and iSeFallED projects collectively as the “study team” throughout the remainder of this manuscript.

### Participants

The study sample consisted of the members of the PRT, six patient representatives and five health service representatives. The patient representatives, three men and three women with a mean age of 69.7 years were persons who have experienced falls themselves. Four of these members knew each other for approximately three years, because they participated in the previous PRT. The health service representatives, four women and one man with an average age of 56.8 years, included persons from organizations which are related to sport clubs or sport associations, senior citizens and care support centers or dementia information and coordination centers. Most interviews were conducted at the private home of the participants. Some of the health services representatives knew each other before participating in the meetings through their own work. The youngest participant of the PRT is 52 years old and the oldest participant is 86 years old. The median age of all participants is 67 years.

### Data Collection

During the PRT meetings, members were provided with detailed information about the planned interviews, including their purpose, and were explicitly informed that participation was voluntary and not a prerequisite for involvement in the PRT. To facilitate understanding, key information was also presented in written form on PowerPoint slides, and participants received a written protocol summarizing essential study details. After allowing sufficient time for reflection, participants provided verbal informed consent. Individual appointments for telephone interviews were subsequently scheduled for early January 2025. At the outset of each interview, the independent interviewer offered a brief introduction outlining the interview procedure, data protection measures, and participants’ rights, including the right to withdraw at any point without consequences. Verbal consent was reconfirmed prior to commencing the interview, and participants’ permission was obtained to initiate audio recording. The interview guide addressed five major thematic areas (see Table 1).

**Table 1:** – Main topics of interview

Topic	Subtopics
Warm-up	How PRT members experienced the first meetings; their own experiences with falls (if applicable); experiences with falls and falls prevention in their professional background; how these experiences contribute to PRT work; and their motivation for participating in the PRT.
Role within research	Benefits of participating, measures to enhance participation
Atmosphere	General atmosphere, atmosphere within discussions, communication with other PRT members, communication with study team, beneficial factors for working together at PRT meetings, missing perspectives in PRT team
Feedback	How individuals feel about their PRT- experience, what works well, what doesn’t?
Knowledge	Take-aways of work within PRT

At the end of the interview participants were given the opportunity to ask questions or raise additional points. Two slightly distinct interview guides were used: one for patient representatives and one for health service representatives. For example, health service representatives were asked about their work background during the warm-up phase, whereas patient representatives were asked about their everyday experience with falls prevention.

To ensure clarity and appropriateness of the questions, multiple feedback loops were conducted within the study team during the development of the interview guides. All interviews were conducted by a female researcher (EMS), who had no prior involvement in PRT meetings. Each interview lasted approximately 30 min. After each interview, EMS wrote a postscript documenting the interview context, including any disturbances, and conversations before or after the official recording.

All interviews were audio-recorded and transcribed verbatim using the KI-based transcription tool of the Software MAXQDA Version 24.7.0 [20]. Transcripts were subsequently proofread and pseudonymized. Audio recordings and transcripts were stored on a secure university data server, accessible only to authorized personnel. Following the completion of the study, all data have been deleted in accordance with the study protocol.

### Analysis

Data of all interviews were analyzed with qualitative content analysis as described by Kuckartz [21] using the Software MAXQDA Version 24.7.0 [20]. Two researchers (LH and EMS) performed the analysis. LH leads and organizes the PRT. Categories were first derived deductively based on the interview guide. Then, both researchers coded the first set of three interviews independently. After discussion between both researchers, a second round of independent coding was started adding inductive codes. Both researchers met again for discussion and adjustment of the coding system until they reached consensus and agreed that the system was substantially developed and appropriate for coding the rest of material. Upcoming disagreements between researchers during this process were dissolved in discussions until consensus was reached. Table 2 shows the coding process, exemplified by one interview sequence.

**Table 2:** Steps of analysis

Meaning Unit	Condensed Meaning Unit	Interpretation of underlying meaning	Subcategory	Main Category
“I think for this kind of research it’s important to leave the ivory tower and develop something that’s practical and solutions that are ready to be used. And for that, you need people like us in the process so that you accommodate the target group.” (PR_06)	The person finds it important that research targets the needs of the community.	PRTs are necessary to make research usable community wide	Making research more applicable	Role in research

The interviews were conducted and analyzed in German. Citations used in this manuscript were translated into American English and reviewed by the research team to ensure a valid translation. Quotations are labeled with the abbreviation PR for patient representatives and HSR for health service representatives, followed by the interview number (e.g. PR_01; HSR_09). Participants did not receive transcripts after the interviews but were given the opportunity to provide additional feedback or raise concerns during subsequent PRT meetings.

This study is reported in accordance with the standards for reporting qualitative research: a synthesis of recommendations (SRQR) and the consolidated criteria for reporting qualitative research (COREQ) [22, 23].

## Results

Our interviews revealed new insights into factors that contribute to successfully planning and conducting participatory research as part of implementing secondary falls prevention. Five primary themes emerged from our analysis that appeared to be of major interest when initiating participatory research in this field (see Table 3):

**Table 3:** Main categories and their subcategories: Key Factors Contributing to Successful Meetings

Main categories	Subcategories
Creating conditions for meaningful participation in research meetings	Structured meetings with clear thematic focus Introductory round at meetings Small-group sessions alternating with plenary discussions Clear presentations with understandable language Written protocols after meetings Regular contact between meetings In-depth introduction into study
Fostering a constructive and inclusive communication climate	Communication between members: - Constructive, respectful - Open dialogue and exchange of perspectives - Ability to make compromises - Diverse professional and personal perspectives Communication between members and study team: - Clear, structured, and comprehensible - trusted and reliable - Communication on equal footing - Attention to individual - Diversity in age contributes to exchange
Active and community facilitating role within the research project	Active, and important Experiences contribute to research PRT members establish connection to locals Science is perceived in a certain way
Bidirectional information exchange throughout the meetings	Patient representatives report on own fall experience Meetings contributed to change on perspective on falls and on higher motivation to be active Knowledge from meetings was used in everyday life
Intrinsic motivation for participation	Intrinsic factors: - helping others through sharing own experiences - staying cognitive and physically fit - exchange with younger people - insights into research and interesting projects Extrinsic factors: - benefits for own organization

### Theme 1) creating conditions for meaningful participation in research meetings

Data analysis highlighted the importance of structured and deliberately organized participatory research meetings as a prerequisite for effective collaboration. Patient representatives and health service representatives emphasized the value of incorporating a brief introductory segment at the beginning of each meeting—such as a check-in or “flashlight round”—which served to establish a shared understanding of the agenda and provide orientation. A clearly defined temporal structure and thematically focused meeting agendas were consistently regarded as essential. This clear structure may provide a sense of safety, which appears to be important for the members. Because many of them have never worked in a PRT before, safety and reassurance may be particularly significant: “*What I really liked was that the meetings with the project team had a clear structure*,* timeline and substantive focus and that you took us and our experiences seriously.” (PR_03)*.

The use of small-group sessions to address specific tasks was perceived as particularly beneficial, as these formats enabled more active engagement. Engagement may be easier in small groups than in larger ones, as they facilitate conversations in which participants can share and compare their experiences. Small groups may be particularly helpful for individuals who do not feel comfortable speaking up in larger groups. The subsequent plenary discussions were considered conducive to integrating multiple perspectives and fostering collective reflection: *“Working in small groups. That I liked. One could tell that it helped us get connected. It really worked in the sense that we as council members got to know each other better.” (HSR_11)*.

PRT members emphasized the importance of presenting topic-specific research content in a clear, understandable manner. From the perspective of the patient and health service representatives, it was regarded as essential that the study team convey complex scientific concepts using plain language within presentations or group discussions. This may enhance participants sense of inclusion. Therefore, the ability to translate scientific information into comprehensible, concise formats was therefore perceived as a key competence for the study team and an important condition for successful collaboration.

In addition to the meetings, continuous communication between sessions, via email or telephone, was described as a crucial element of participatory collaboration. This applied both to interactions among patient and health service representatives and to exchange with the research team. Such communication was seen as supporting transparency and contributing to the development of trust and sustained cooperation. Regular consultation may enhance the feeling of participants to be valuable for the research project.

The systematic preparation of written protocols by the study team was also highlighted as important. This not only facilitated further communication but also served as a basis for clarifying follow-up tasks. Written protocols may also serve as a resource for members to prepare for subsequent meetings. Finally, respondents emphasized the importance of allocating sufficient time at the beginning of the research process for an in-depth introduction to the study, thereby ensuring a shared conceptual and contextual understanding among all PRT members.

### Theme 2) fostering a constructive and inclusive communication climate

Communication within PRT meetings can be differentiated into two different levels: [1] communication among the PRT members themselves, primarily patient and health service representatives, and [2] communication between the study team and the PRT members.

#### [1] communication among the PRT members themselves, primarily patient and health service representatives

Patient and health service representatives consistently described communication among PRT members as constructive and respectful. A recurring theme was the perception of a safe and supportive space that enabled open dialogue and the exchange of differing perspectives. A fall can be an experience that is connected with negative emotions; therefore, an open atmosphere that allows individuals to share these experiences without being judged is important. As one PRT member noted about the communication: “*Absolutely*,* it was communication among equals. We all listened*,* no one interrupted*,* like normal interactions with others.” (HSR_08)* This positive interpersonal exchange was considered fundamental for fostering trust and active participation.

Furthermore, beyond the importance of a secure and inclusive atmosphere, participants emphasized the need for meetings to result in a tangible outcome or consensus. Even when discussions were controversial or involved divergent opinions, patient and health service representatives valued the group’s ability to reach acceptable compromises. Reaching such shared understandings was regarded as a key indicator of successful collaboration. As it was stated: *“I think the exchange is extremely valuable. That we talk about all these topics in a constructive way and that we are on the same page.” (HSR_10)*.

In addition to being able to make compromises, the individual experiences and backgrounds of PRT members were valued as important resources. The diverse professional and personal perspectives brought into the process were seen as enriching the discussions and expanding the collective understanding of the research topic. Combining participants with personal experiences of falls and those who approach the topic from a professional perspective may broaden perspectives. As emphasized: “*You can tell there’s a lot of experience*,* showing a perspective that not everyone would be aware of. For me*,* the contributions had important take aways.” (PR_04)*.

This appreciation for personal and professional experience highlights the importance of heterogeneity within participatory groups, not only for representation but also for substantive contribution.

#### [2] Communication between the study team and the PRT members

The communication between the study team and the members of the PRT was consistently described as positive and constructive. PRT members emphasized that the study team communicated in a clear, structured, and comprehensible manner, particularly when conveying complex or technical content: “*I can only repeat*,* pleasant*,* personal*,* and when it came to the content*,* very elaborate and well structured*,* and clear in the way the information was presented*,* so that we could follow easily.” (PR_03)*.

This clarity and transparency were seen as fundamental to enabling meaningful engagement with the study content.

In addition to the comprehensibility of the information, trust was identified as a crucial factor in communication. Members of the PRT stressed the importance of having a study team who foster a sense of trust and reliability, particularly when engaging in more complex or sensitive discussions. The ability to create a communicative atmosphere that was not only respectful but also empowering was considered a strength of the study team.

Equally important was the perception that communication occurred on equal footing. Members of the participatory research team appreciated that their perspectives were treated as valuable and that discussions with the study team took place at eye level. Moreover, feedback on the integration of developed topics was perceived as reinforcing the participatory character of the collaboration and contributed to a sense of shared responsibility and mutual respect within the research process.

Moreover, participants emphasized the importance of a welcoming and appreciative atmosphere within the participatory research meetings. Interpersonal exchange and attention to individual well-being were seen as central components of a successful collaboration. Starting the sessions by giving everyone the time to settle in and to participate : “*Each session starts with a general check-in*,* along the lines of how are you doing today*,* and what was going on in the past weeks*,* and how are you all personally? … I enjoy being there and always like to come back. And that’s the spirit of the situation and of this team.” (PR_03)*.

This kind of emotional attunement was interpreted as a sign of appreciation and respect, contributing to a positive group dynamic and a sense of belonging to the group.

In addition, the age and life experience of the PRT members were seen as contributing positively to the group atmosphere. The diversity in age was not regarded as a challenge but rather as an asset, particularly because older participants brought a wealth of personal and professional experience to the discussions: “*We really get along. I have to say it’s a nice mixture of experiences*,* the older members are over 70 and can contribute their experience with aging.” (PR_05)* Another health service member also perceived the diversity as positive: *“So*,* having all these different ages in the group is great.“ (HSR_07)*.

These reflections suggest that the inclusive, respectful handling of different generational perspectives was a key aspect in creating a supportive and collaborative meeting atmosphere.

Overall, participants valued not only the opportunity to contribute to the content of the study, but also the social and emotional dimensions of their involvement. This combination of content-oriented work and genuine personal exchange appeared to strengthen long-term engagement and fostered a cohesive and motivated advisory group.

### Theme 3) active and community facilitating role within the research project

Patient representatives and health service representatives perceived their role within the PRT as active and important. In comparison to patient representatives, health service representatives described their role as more consultative and acknowledged that the study team was bearing most of the responsibilities. Both groups believed that they can contribute their experiences to the PRT meetings.

The participants described themselves as important persons to establish connections between the study team, and local organization. These local connections support community-based falls prevention efforts. They also mentioned the value of the PRT in combining theory and practice and therefore, making research more applicable for the community: *“I think for this kind of research it’s important to leave the ivory tower and develop something that’s practical and solutions that are ready to be used. And for that*,* you need people like us in the process so that you accommodate the target group.” (PR_06)*.

Interviewees further reflected on their role in falls prevention research. Participants viewed themselves as distinct from the research team, whom they perceived as holding specialized knowledge and expertise that was not always accessible to the average person. However, the PRT meetings seem to have a positive influence on bringing both groups closer together: “*The disconnect between scientists and us “normal people” is really not that big.” (PR_01)*.

Participants highlighted several factors that helped bring these groups closer together. In particular, they appreciated that the entire team worked collaboratively and shared practical tasks, such as cleaning up after meetings, which reduced perceived hierarchies. Patient and health service representatives stated that they needed some time to get used to research, because of their lack of prior experiences. A patient representative said: “*I was totally unfamiliar with this. My sense was that I have to get used to it*,* because it really is a new world for me.” (PR_02)*.

Participants also described a university-typical language, including the use of foreign words or “scientific terminology“ *(PR04)* that could be at times experienced as alienating. As mentioned earlier, the members of the study team were acknowledged for providing a level environment and using understandable language: *“So*,* I think when you work in academia you live with a certain language style and then in the council different languages need to come together*,* and you all handle that really well.” (PR_01)*.

### Theme 4) bidirectional information exchange throughout the meetings

Within the meetings, participants contributed their knowledge to the meetings, but were also able to take away knowledge, which they then discussed in their private or work life. This bidirectional knowledge exchange was reported by patient representatives and health services representatives.

Patient representatives reported mostly on their own fall experience or the fall experiences of relatives, explaining how the falls happened and the consequences of these falls, such as concerns or hospital stays. Participating in the PRT led to a more differentiated perspective on falls, such as being more conscious of the topic falls. It also led to a higher motivation to be physical active in everyday life to potentially prevent falls. The participants appreciated that both studies provide secondary prevention measures after a falling event. Patient representatives described this experience as useful for taking on the perspectives of potential study participants. The topic “falls” was mentioned predominantly by patient representatives.

Health services representatives reported more from their work experience in sports clubs or their work as consultants for older adults: *“I was able to compare my experiences. In my work context I am thinking about what’s missing*,* what works*,* what could be helpful. So*,* what I share in the council comes from my real-life experiences. As you said theory versus real life are often far apart and to help connect the two that is really exciting.” (HSR_08)*.

While both groups shared different knowledge themes, both reported the value from the experiences gained through participating in the meetings. Participants noted that the opportunity to take part in a lecture, which was organized through the study team, was very beneficial. Another important takeaway the participants added was getting insights into research. The participants also believed that they will gain practical experience through trying out the perturbation treadmill, which was planned after these interviews. Getting in contact with other people was deemed a positive experience from patient and health service representatives. They described the positive feeling resulting from participating in the PRT as finding meaning and being able to actively participate in something bigger: *“Participating in the council gave me a sense of making an active contribution. So*,* I contribute to society in a way that’s meaningful to me and that makes me feel good.“ (PR_06)* The bidirectional exchange of knowledge and experiences during meetings allows not only individuals to gain knowledge but also their peers, other PRT members, and the study team.

### Theme 5) intrinsic motivation for participation

Motivation emerged as a central mechanism underpinning the quality and depth of collaboration within the participatory research process. Patient representatives primarily described intrinsic motivational drivers, including enjoyment of participation, opportunities to remain cognitively and physically active, and the value of exchanging perspectives with younger researchers involved in the project. These aspects were perceived as personally meaningful and helped sustain engagement over time. Participants reported that attending the meetings was beneficial for maintaining cognitive and physical activity. In addition, the interaction with younger members of the research team was described as particularly engaging. Beyond knowledge exchange, the PRT meetings were perceived as providing cognitive stimulation and meaningful dialogue, which participants considered especially valuable in face-to-face settings. As one participant explained: “And because I have put a lot of thought into these things I could make an important contribution and that motivates me to stay active and connect with younger people. That is a particularly nice part of this work at the university, that I have a chance to hang out with young people.” (PR_06) One representative also noted being impressed by the enthusiasm and proactive contributions of others, describing this as an indicator of the strong motivation driving participation. Participants valued the PRT not only for the input they received but also for the opportunity to contribute in ways that could benefit other patients with lived experience as well as the study team. This motivation to support individuals with similar experiences may stem from feeling left alone after a fall and knowing that the current support system is not sufficient, as well as from experiencing the consequences of a fall. While some expectations, such as higher participation rates for the sport club could not be fulfilled, the other intrinsic motivational aspects seem to have compensated for the time and effort participants invest in attending the meetings: *“It’s always a lot of work but I think it’s totally worth it*,* to take and to make the time for it.” (HSR_09)* Furthermore, the general high and mostly intrinsic motivation led to a high level of engagement and commitment during meetings.

Importantly, intrinsic motivation did not merely facilitate attendance but shaped the intensity and quality of engagement. Participants who experienced the meetings as meaningful reported investing effort in preparation and contributing actively to discussions, thereby enhancing the deliberative character of the group. Although some extrinsic expectations, such as higher participation rates in the sport club, were not fully met, intrinsic drivers appeared to buffer potential frustration and supported continued commitment. Overall, the interplay between intrinsic satisfaction and externally oriented goals influenced sustained participation, depth of contribution, and collaborative group dynamics within the PRT.

## Discussion

We found several important facilitators of conducting participatory research for falls prevention in our interviews, such as methods that enable exchange, clear communication on eye level, an active role with possibilities to contribute experiences and to gain knowledge, as well as intrinsic motivation. Rather than representing isolated facilitation techniques, these elements collectively functioned as structural conditions that enabled meaningful collaboration and shaped the quality of interaction within the PRT. While individuals with lived experience and professional stakeholders perceived the participatory research approach similarly overall, they differed slightly in their motivations for engaging in such research.

### Creating conditions for meaningful participation in research meetings

Participants in the PRT described themselves as a team and highlighted that methods such as flashlight rounds or discussions in smaller groups were particularly effective in fostering a sense of togetherness. In line with previous research, small group formats enabled participants to get to know one another better and engage in more in-depth exchanges of perspectives as patient or health service representatives [24]. Our findings extend this literature by illustrating how such formats not only enhance relational closeness but also facilitate epistemic exchange, allowing experiential and professional knowledge to intersect more productively within falls prevention research. Consequently, the development of heterogenous PRTs may benefit from incorporating such methods and intentionally allowing space for informal, non-professional interaction to support team building.

In this specific study, four members had already known each other through their participation in a previous PRT. To bridge the gap between longstanding and newly recruited participants, rotating small group exchanges further enhanced the ability to work together and positively influence group dynamics. Newer participants described the interaction with “long-standing” members as beneficial, as it allowed for mutual learning while diminishing the divide between old and new members. Working in small groups especially supported a sense of connection – even after just four months of collaboration. This suggests that structured rotation mechanisms may actively mitigate hierarchies of experience within participatory teams, thereby supporting more equitable contribution. Thus, this format may also be advantageous when, for example, changes in PRT composition occur due to dropouts, as it can facilitate the integration of new members despite differing levels of prior knowledge.

In addition to small group methods, presentations were considered important for conveying project-related information. These presentations should be designed clearly and easy to understand [25]. Therefore, a combination of small group sessions and well-prepared presentations may enhance the quality of exchange within the meetings. Taken together, our findings suggest that balancing information provision with interactive formats may represent a key design principle for participatory structures in falls prevention research, where both technical knowledge and lived experiences need to be integrated. Future research could further investigate the ideal ration between presentations and methods, in which active exchange is possible for PRTs.

### Fostering a constructive and inclusive communication climate

The emotional and interpersonal aspects of the meetings played a central role in fostering commitment. Interest for one another and a positive culture within a team were factors that may foster trust [26]. Open and respectful dialogue offered the chance to learn about other views and see that experiences may be common, which may additionally enhance the feeling of trust in participatory research teams [27]. Another facilitator under the theme of communication was giving attention to everyone, for example through check-ins at the beginning of meetings [28].

Rather than being peripheral soft factors, these elements appeared to function as core enabling mechanisms for sustained collaboration. Participants highlighted the welcoming atmosphere, and mutual appreciation as essential contributors to a strong sense of belonging. This sense of belonging, in turn, was closely linked to their willingness to voice personal experiences and critical reflections, an essential prerequisite in falls prevention research, where discussions often involve vulnerability, concerns about falling, and perceived loss of independence.

As the building of trust is not a linear relationship and has to be maintained over time, our findings support existing theoretical models that conceptualize participatory collaboration as a developmental process rather than a static state [5]. Our findings suggest that deliberate communicative practices, such as structured check-ins and explicit appreciation, may accelerate the transition from initial orientation to more cohesive and productive collaboration. Our findings extend prior literature by demonstrating how specific facilitation techniques operationalize concepts such as trust and inclusivity within an ongoing PRT structure. Future research may further examine how these emotional dynamics evolve longitudinally and how they influence knowledge integration and decision-making processes.

Another contributor to creating a positive atmosphere was the composition of the group. While the patient representatives were predominantly older adults, the research team consisted mainly of younger researchers. This age diversity was noticed and appreciated by participants, who described the exchange across generations as enriching. Previous research suggests that intergenerational interaction can foster mutual understanding and broaden perspectives. Moreover, older adults have reported positive emotions such as contentment and confidence, and younger adults benefit from developing both soft and hard skills [29]. When examining diverse teams more broadly, the exchange of skills and new perspectives has been highlighted as a key strength [30]. Our findings nuance this literature by showing that age diversity did not introduce generational tensions, despite the topic primarily affecting older adults. Instead, respectful communication and shared goals appeared to mitigate potential hierarchical or generational divides. This is particularly relevant in falls prevention research, where interventions target older populations but are often developed and implemented by younger professionals. Intentional inclusion of diverse age groups, or at minimum, diverse experiential perspectives, may, therefore, strengthen mutual understanding and enhance the relevance and acceptability of preventive strategies [31, 32].

### Active and community facilitating role within the research project

Generally, participants seemed to be unfamiliar with science and sometimes held preconceptions about it, such as perceiving science as something distant or elitist (e.g., “in the ivory tower”). Actively involving people in research projects may help make science more accessible and understandable, both in general and for specific target groups [33]. Our findings suggest that participation in the PRT did not merely increase knowledge about a specific project but contributed to a shift in how research itself was perceived transforming it from a distant authority into a collaborative and approachable process. In the context of current societal challenges, such participatory approaches may also contribute to building bridges between researchers and the public and thereby support more transparent and accessible science communication.

Building connections between researchers and participants thereby plays a crucial role, as these relationships can reduce perceived barriers and make research more approachable. This relational proximity appeared to position PRT members not only as contributors within the project but also as informal ambassadors of the research within their communities. Participants described sharing newly acquired knowledge about falls prevention with peers and within local networks, suggesting that participatory structures may foster community-level diffusion of evidence-based information.

This finding extend existing participatory research literature by highlighting a community-facilitating function of structured PRTs within falls prevention. While many participatory approaches emphasize co-design and shared decision-making, less attention has been paid to how ongoing PRT engagement may enhance knowledge translation beyond the immediate research setting. In a field such as falls prevention, where strong evidence for effective interventions exists but uptake and adherence remain limited, such community-embedded dissemination mechanisms may be particularly valuable. However, the long-term impact of this community-facilitating role remains to be systematically evaluated. As an outlook, the true added value of PRTs will ultimately need to be assessed in relation to implementation outcomes, such as reach, adoption, and sustained engagement in preventive programs. Addressing this will require methodologically robust and adequately funded evaluation strategies capable of capturing indirect and community-level effects of participatory involvement.

### Bidirectional information exchange throughout the meetings

Patient representatives actively contributed tacit knowledge by sharing personal fall experiences, which they also identified as a key motivational factor. In turn, they acquired new insights during the participatory process that shifted their perspectives on falls and were applied in daily life [34]. This reciprocal exchange illustrates that the PRT functioned not only as a consultative body but as a site of mutual learning, in which experiential and scientific knowledge were continuously negotiated and integrated.

Facilitating experience-sharing within PRTs may therefore improve the translation of tacit knowledge into research, particularly relevant in areas such as falls prevention, where evidence-based interventions exist, but implementation and reach to those who could benefit most remain inadequate [35]. Our findings suggest that integrating lived experience into ongoing deliberation processes may help contextualize established falls prevention strategies, making them more responsive to perceived barriers, concerns, and everyday realities of older adults.

Importantly, the exchange was not unidirectional. While researchers gained insight into the lived experience of those, who have experienced falls before, patient representatives reported modifying their own attitudes and preventive behaviors based on scientific input received during meetings. This dynamic highlights a dual translational pathway: from community to research (informing intervention development) and from research back to community (informing everyday preventive practices).

By explicitly examining this bidirectional mechanism within a structured and regularly meeting PRT, our study contributes knowledge to participatory research literature, which often emphasizes co-design but less frequently analyzes how ongoing dialogue shapes knowledge integration over time. In the context of falls prevention, where adherence to preventive exercise and behavioral recommendations remain low, such sustained knowledge exchange may represent a critical mechanism for strengthening both intervention relevance and community uptake.

### Intrinsic motivation for participation

Notably, motivational factors differed somewhat between participant groups. While patient representatives emphasized helping others and staying mentally active, health service representatives more often mentioned external benefits, such as potential advantages for their organizations. This differentiation suggests that participatory engagement is sustained by a constellation of intrinsic and extrinsic drivers, rather than by a uniform motivational profile. Recognizing and addressing these different motivational drivers can help in designing more inclusive and sustainable participation structures. Similar findings were reported in a systematic review [15], in which older participants also described feelings of being valued [36], gaining knowledge [37], increased confidence, and challenging stereotypes [38]. While prior research has primarily documented these motivational benefits as outcomes of participation, our findings indicate that intrinsic motivation may also function as a stabilizing mechanism within ongoing participatory structures. In our study, participants continued to engage actively even when certain anticipated external outcomes, such as increased participation in affiliated programs, were not fully realized. This suggests that perceived meaningfulness and the opportunity to contribute to the well-being of peers with lived experience of falls may buffer potential disappointment related to limited measurable impact. Particularly in falls prevention, where behavioral change and implementation processes are often slow and incremental, sustaining engagement through intrinsic commitment may be critical for long-term collaboration. By highlighting how motivational dynamics may shape both participation continuity and contribution quality, this study extends existing literature beyond descriptive accounts of participant satisfaction toward a more process-oriented understanding of engagement in falls prevention research. However, given the cross-sectional nature of the data, the influence of motivational factors on sustained participation over time warrants further longitudinal investigation. Understanding and addressing the diverse motivational drivers of different participant groups is crucial for building inclusive and sustainable participation structures, as these motivations shape engagement and the benefits participants experience.

### Strengths, limitations, and future directions

Conducting interviews proved to be a valuable method for capturing the experiences and perspectives of both patient and health service representatives. A key strength of this study is that the entire PRT participated, ensuring comprehensive coverage of all group members. Moreover, several participants reported similar observations, suggesting thematic saturation in areas such as motivation and atmosphere. Achieving saturation is considered an important criterion in qualitative research, as it indicates that perspectives have been sufficiently represented and that the findings provide a robust reflection of participants’ views.

One limitation of this study was that only PRT members were interviewed, not the research team. While the interview guideline was explicitly designed for participants, including the researchers’ perspectives could have enriched the analysis by enabling comparisons between both sides of the process. Such an approach would, however, require external supervision to minimize bias, which was not feasible in the present study. Nevertheless, future research should consider capturing all perspectives, as this could provide a more comprehensive understanding of participatory dynamics and the development of PRTs. Achieving this would require adequate methodological and financial resources to support the additional effort.

In line with this, future studies might also explore how perspectives and group dynamics evolve throughout the lifecycle of PRTs, and how engagement shapes participants’ understanding and perception of the research process. Particular attention could be directed toward the interplay between emotional connectedness, reciprocal knowledge exchange, and motivational drivers, as these factors collectively influence the long-term sustainability and practical impact of participatory research in public health.

A related challenge remains the difficulty of objectively measuring the true impact of PRT involvement. While integrating patient perspectives is clearly valuable, robust evaluation of the added benefit for implementation is hindered by the lack of feasible study designs that would allow direct comparison, i.e., conducting identical research simultaneously with and without PRT involvement, a scenario rarely possible in practice. Addressing this challenge in future studies would require additional methodological and financial support, highlighting the need for dedicated resources to fully assess the practical impact of participatory research in falls prevention.

## Conclusion

Our study provides valuable insights into key factors that facilitate successful participatory research in the context of secondary falls prevention. Structured meeting formats, clear and respectful communication on equal footing, and the active inclusion of participants’ personal and professional experiences emerged as central components for effective collaboration. Participatory methods, such as small group discussions, check-ins at the beginning of meetings, and the use of accessible language, fostered not only meaningful exchange but also a strong sense of team cohesion among participants. Findings further highlight that intrinsic motivation in order to maintain cognitive and physical activity, played a crucial role in forming active engagement. Participation in the PRT was not only experienced as personally meaningful but also created opportunities for participants to share newly acquired knowledge in their everyday social and professional environments, thus reinforcing a reciprocal flow of information. Overall, this study highlights the potential of participatory approaches to make research processes more accessible and practice oriented. Further research is needed to explore how roles and perceptions within participatory research teams evolve over time and how participatory approaches can contribute to more targeted and effective health service research.

## Supplementary information

Below is the link to the electronic supplementary material.

Supplementary Material 1

## Data Availability

The dataset used and analyzed during the current study will be made available by the corresponding author upon reasonable request. As public availability would compromise patient privacy, storing the data in a public repository is not possible. However, upon request and after approval by the local ethics board, it may be made available. To receive data, please contact the corresponding author.

## Abbreviations

COREQ: consolidated criteria for reporting qualitative research
HSR: health service representatives
iSeFallED: “Tailored Interventions to Prevent Functional Decline After a Sentinel Fall Presenting to the Emergency Department – iSeFallED
PPI: patient and public involvement
PR: patient representatives
PRT: participatory research teams
SRQR: standards for reporting qualitative research: a synthesis of recommendations
TRAIL: Training at the Limit of Balance Control on a Perturbation Treadmill to Prevent Unrecovered Falls in Geriatric Patients With and Without Cognitive Impairment
