# Supplementary material for: Building diverse participatory research teams in falls prevention: a qualitative interview study
Source: BMC Geriatr. 2026 Nov 10;26:1403. doi: 10.1186/s12877-026-08686-y (PMC13753275; doi:10.1186/s12877-026-08686-y)
Supplement: Supplementary file 1 [file 12877_2026_8686_MOESM1_ESM.docx]

Consolidated criteria for reporting qualitative studies (COREQ): 32-item checklist

| **No** | **Item** | **Guide questions/description** |  |
| --- | --- | --- | --- |
| **Domain 1: Research team and reflexivity** |  |  |  |
| Personal Characteristics |  |  |  |
| 1. | Interviewer/facilitator | Which author/s conducted the interview or focus group? | “All interviews were conducted by a female researcher (EMS), who had no prior involvement in PRT meetings” (p.5) |
| 2. | Credentials | What were the researcher's credentials? *E.g. PhD, MD* | / |
| 3. | Occupation | What was their occupation at the time of the study? | See Affiliations |
| 4. | Gender | Was the researcher male or female? | “All interviews were conducted by a female researcher (EMS), who had no prior involvement in PRT meetings” (p.5) |
| 5. | Experience and training | What experience or training did the researcher have? | / |
| Relationship with participants |  |  |  |
| 6. | Relationship established | Was a relationship established prior to study commencement? | “All interviews were conducted by a female researcher (EMS), who had no prior involvement in PRT meetings” (p.5) |
| 7. | Participant knowledge of the interviewer | What did the participants know about the researcher? e*.g. personal goals, reasons for doing the research* | “All interviews were conducted by a female researcher (EMS), who had no prior involvement in PRT meetings” (p.5) |
| 8. | Interviewer characteristics | What characteristics were reported about the interviewer/facilitator? e.g. *Bias, assumptions, reasons and interests in the research topic* | “LH leads and organizes the PRT” (p.6) |
| **Domain 2: study design** |  |  |  |
| Theoretical framework |  |  |  |
| 9. | Methodological orientation and Theory | What methodological orientation was stated to underpin the study? *e.g. grounded theory, discourse analysis, ethnography, phenomenology, content analysis* | “Data of all interviews from both groups were analyzed using qualitative content analysis as described by Kuckartz [13].” (p.6) |
| Participant selection |  |  |  |
| 10. | Sampling | How were participants selected? *e.g. purposive, convenience, consecutive, snowball* | “We conducted eleven semi-structured telephone interviews with each member of a PRT.” (p. 3) |
| 11. | Method of approach | How were participants approached? e*.g. face-to-face, telephone, mail, email* | “Within one of the PRT-meetings the team was informed about the interviews and asked for consent to participate. Once they gave consent, appointments for telephone interviews were scheduled for the beginning of January 2025.“ (p.4) |
| 12. | Sample size | How many participants were in the study? | “The study sample consisted of the members of the PRT, six patient representatives and five health service representatives.” (p.3) |
| 13. | Non-participation | How many people refused to participate or dropped out? Reasons? | “We conducted eleven semi-structured telephone interviews with each member of a PRT.” (p. 3) |
| Setting |  |  |  |
| 14. | Setting of data collection | Where was the data collected? e*.g. home, clinic, workplace* | “Most interviews were conducted at the private home of the participants.” (p.4) |
| 15. | Presence of non-participants | Was anyone else present besides the participants and researchers? | / |
| 16. | Description of sample | What are the important characteristics of the sample? *e.g. demographic data, date* | “The patient representatives, three men and three women with a mean age of 69.7 years, were persons who have experienced falls themselves. The health service representatives, four women and one man with an average age of 56.8 years, included persons from organizations which are related to sport clubs or sport associations, senior citizens and care support centers or dementia information and coordination centers.” (p.3-4) |
| Data collection |  |  |  |
| 17. | Interview guide | Were questions, prompts, guides provided by the authors? Was it pilot tested? | “The interview guide targeted five major thematic areas (SEE TABLE 1).” (p.4)  “To ensure clarity and appropriateness of the questions, multiple feedback loops were conducted within the study team during the development of the interview guides.” (p.5) |
| 18. | Repeat interviews | Were repeat interviews carried out? If yes, how many? | N/A |
| 19. | Audio/visual recording | Did the research use audio or visual recording to collect the data? | “All interviews were audio-recorded and transcribed.” (p.6) |
| 20. | Field notes | Were field notes made during and/or after the interview or focus group? | “EMS wrote a postscript documenting the interview context, including any disturbances, and conversations before or after the official recording.” (p.6) |
| 21. | Duration | What was the duration of the interviews or focus group? | “Each interview lasted approximately 30 minutes.” (p.5) |
| 22. | Data saturation | Was data saturation discussed? | „Moreover, several participants reported similar observations, suggesting thematic saturation in areas such as motivation and atmosphere. Achieving saturation is considered an important criterion in qualitative research, as it indicates that perspectives have been sufficiently represented and that the findings provide a robust reflection of participants’ views. “ (p.20) |
| 23. | Transcripts returned | Were transcripts returned to participants for comment and/or correction? | “Participants did not receive transcripts after the interviews but were given the opportunity to provide additional feedback or raise concerns during subsequent PRT meetings.” (p.7) |
| **Domain 3: analysis and findings** |  |  |  |
| Data analysis |  |  |  |
| 24. | Number of data coders | How many data coders coded the data? | “Two researchers (LH and EMS) performed the analysis.” (p.6) |
| 25. | Description of the coding tree | Did authors provide a description of the coding tree? | / |
| 26. | Derivation of themes | Were themes identified in advance or derived from the data? | “A category frame was developed inductively, and the analysis process was iterative. Both researchers independently coded the interviews.” (p.6) |
| 27. | Software | What software, if applicable, was used to manage the data? | “Data analysis was supported using MAXQDA 24.7.0 [14].” (p.7) |
| 28. | Participant checking | Did participants provide feedback on the findings? | / |
| Reporting |  |  |  |
| 29. | Quotations presented | Were participant quotations presented to illustrate the themes / findings? Was each quotation identified? e*.g. participant number* | e.g. „A clearly defined temporal structure and thematically focused meeting agendas were consistently regarded as essential: “*What I really liked was that the meetings with the project team had a clear structure, timeline and substantive focus and that you took us and our experiences seriously.” (PR_03)” (p.9)* |
| 30. | Data and findings consistent | Was there consistency between the data presented and the findings? | “Generally, participants seemed to be unfamiliar with science and sometimes held preconceptions about it, such as perceiving science as something distant or elitist (e.g., "in the ivory tower").” (p.20) |
| 31. | Clarity of major themes | Were major themes clearly presented in the findings? | “Five primary themes emerged from our analysis that appeared to be of major interest when initiating participatory research in this field (see table 3):” (p.7) |
| 32. | Clarity of minor themes | Is there a description of diverse cases or discussion of minor themes? | “Notably, motivational factors differed somewhat between participant groups. While patient representatives emphasized helping others and staying mentally active, health service representatives more often mentioned external benefits, such as potential advantages for their organizations.” (p.21) |

Tong A, Sainsbury P, Craig J. Consolidated criteria for reporting qualitative research (COREQ): a 32-item checklist for interviews and focus groups. International Journal for Quality in Health Care. 2007. Volume 19, Number 6: pp. 349 – 357
